# Digital biomarkers: 3PM approach revolutionizing chronic disease management — EPMA 2024 position

**DOI:** 10.1007/s13167-024-00364-6

**Published:** 2024-05-11

**Authors:** Ivica Smokovski, Nanette Steinle, Andrew Behnke, Sonu M. M. Bhaskar, Godfrey Grech, Kneginja Richter, Günter Niklewski, Colin Birkenbihl, Paolo Parini, Russell J. Andrews, Howard Bauchner, Olga Golubnitschaja

**Affiliations:** 1 University Clinic of Endocrinology, Diabetes and Metabolic Disorders, Skopje, North Macedonia; 2 Faculty of Medical Sciences, University Goce Delcev, Stip, North Macedonia; 3 Veteran Affairs Capitol Health Care Network, Linthicum, MD USA; 4 University of Maryland School of Medicine, Baltimore, MD USA; 5 Endocrinology Section, Carilion Clinic, Roanoke, VA USA; 6 Virginia Tech Carilion School of Medicine, Roanoke, VA USA; 7 Department of Neurology, Division of Cerebrovascular Medicine and Neurology, National Cerebral and Cardiovascular Centre (NCVC), Suita, Osaka Japan; 8 Department of Neurology & Neurophysiology, Liverpool Hospital, Ingham Institute for Applied Medical Research and South Western Sydney Local Health District, Sydney, NSW Australia; 9 NSW Brain Clot Bank, Global Health Neurology Lab & NSW Health Pathology, Sydney, NSW Australia; 10 Department of Pathology, Faculty of Medicine & Surgery, University of Malta, Msida, Malta; 11 CuraMed Tagesklinik Nürnberg GmbH, Nuremberg, Germany; 12 Technische Hochschule Nürnberg GSO, Nuremberg, Germany; 13 University Clinic for Psychiatry and Psychotherapy, Paracelsus Medical University, Nuremberg, Germany; 14 Department of Neurology, Massachusetts General Hospital, Harvard Medical School, Boston, MA USA; 15 Cardio Metabolic Unit, Department of Medicine Huddinge, and Department of Laboratory Medicine, Karolinska Institute, and Medicine Unit of Endocrinology, Theme Inflammation and Ageing, Karolinska University Hospital, Stockholm, Sweden; 16 Nanotechnology & Smart Systems Groups, NASA Ames Research Center, Aerospace Medical Association, Silicon Valley, CA USA; 17 Boston University Chobanian & Avedisian School of Medicine, Boston, MA USA; 18 Predictive, Preventive and Personalized (3P) Medicine, University Hospital Bonn, Rheinische Friedrich-Wilhelms-Universität Bonn, Bonn, Germany

**Keywords:** Digital biomarkers, Non-communicable chronic disease, Health risk assessment, Health-to-disease transition, Health protection, Health economy and policy, Artificial intelligence, Machine learning, Wearable point-of-care devices, Innovative ecosystem, Sleep disorders, Diabetes, Cardiovascular diseases, Chronic obstructive pulmonary disease, Cancer, Perinatal asphyxia, Predictive Preventive Personalized Medicine, PPPM / 3PM, Primary and secondary care

## Abstract

Non-communicable chronic diseases (NCDs) have become a major global health concern. They constitute the leading cause of disabilities, increased morbidity, mortality, and socio-economic disasters worldwide.

Medical condition-specific digital biomarker (DB) panels have emerged as valuable tools to manage NCDs. DBs refer to the measurable and quantifiable physiological, behavioral, and environmental parameters collected for an individual through innovative digital health technologies, including wearables, smart devices, and medical sensors. By leveraging digital technologies, healthcare providers can gather real-time data and insights, enabling them to deliver more proactive and tailored interventions to individuals at risk and patients diagnosed with NCDs.

Continuous monitoring of relevant health parameters through wearable devices or smartphone applications allows patients and clinicians to track the progression of NCDs in real time. With the introduction of digital biomarker monitoring (DBM), a new quality of primary and secondary healthcare is being offered with promising opportunities for health risk assessment and protection against health-to-disease transitions in vulnerable sub-populations. DBM enables healthcare providers to take the most cost-effective targeted preventive measures, to detect disease developments early, and to introduce personalized interventions. Consequently, they benefit the quality of life (QoL) of affected individuals, healthcare economy, and society at large.

DBM is instrumental for the paradigm shift from reactive medical services to 3PM approach promoted by the European Association for Predictive, Preventive, and Personalized Medicine (EPMA) involving 3PM experts from 55 countries worldwide. This position manuscript consolidates multi-professional expertise in the area, demonstrating clinically relevant examples and providing the roadmap for implementing 3PM concepts facilitated through DBs.

## Preamble

A limited access to diagnostic tests is recognized as one of the major barriers to provide healthcare services in remote and low- and middle-income countries (LMIC). In high-income countries (HIC), similar issues arise due to over-burdened and centralized healthcare systems that cannot provide diagnoses in a timely manner. The development of sensitive and accurate smart biosensors has been the focus of a major effort over the last decade to set up point-of-care tests supporting decentralization of healthcare services. Development of portable biosensors require a biorecognition element (BRE) that measures and quantifies the analyte of interest, a transducer that senses a change brought about by the binding of the analyte to the BRE and amplifies this signal so that it is detected by a smart device.

The evolution of wearable point-of-care devices for measuring disease-specific analytes has progressively utilized minimally invasive BREs to detect biomarkers in easily accessible biological fluids including the use of smart contact lenses for tear-based biosensors, epidermal biosensors, oral biosensors to detect salivary metabolites, urinary biosensors, breath-based biosensors, and smartphone-assisted colorimetric biosensors. Specifically in the area of cancer research, various BREs were developed to detect biological makers at physiological ranges, including carcinoembryonic antigen (CEA) detection in saliva showing promise for early oral cancer detection [1], measuring inflammation markers [2], detection of breast cancer marker Mucin1 in serum samples [3], and volatile organic compounds in breath-based biosensors [4] and biological fluids to detect cancers using odorant receptor-based biosensors [5]. Extensive work is being performed in the field utilizing biosensors for lung cancer detection, including the detection of analytes such as circulating tumor nucleic acids including Epidermal Growth Factor Receptor (EGFR) [6] and KRAS [7] mutations, microRNA [8], and specific proteins such as CEA [9].

Advances in microfluidics and nanotechnology provide the platforms towards real-time sensing of molecules and transduce the amplified signal to a digitalized readout, exemplified by personal glucose sensors that relay the measured levels to a smart watch. The enabling technology between the BREs that measure the analyte and readout involve microfluidics and highly sensitive detection systems such as smart devices. The microfluidic devices are low-volume chambers and channels, also known as lab-on-chip. The use of microfluidic point-of-care diagnostic devices coupled with paper-based biosensors and optical smartphone colorimetric readout is exemplified by detection of Alpha-fetoprotein (AFP) and Mucin-16 (MUC16) in serum considered potent biomarkers for liver and ovarian cancers [10], and Prostate-Specific Antigen (PSA) for prostate cancer [11]. Alternatively, the use of electrochemical biomedical sensors are exemplified by Human epididymis protein 4 (HE4) in serum as biomarker for ovarian cancer [12], and detection of tyrosinase (TYR) enzyme as a biomarker to monitor melanomas [13]. The electrochemical sensor measures a differential current following the conversion of the substrate immobilized on the transducer surface when it meets the analyte. The wearable sensors transmit data wirelessly to a smart device through an ultralight and flexible electronic board.

Integration of Artificial Intelligence (AI) with wearable sensors allows monitoring of an individual’s health in real time. The main objective is to transform sensor signals into clinically relevant parameters that improve the quality of care and disease management. The resulting parameters can further be utilized in dedicated AI models to predict disease risk, make diagnoses, and monitor disease progression [14–18].

The main challenge in developing point-of-care diagnostic biosensors is to lower the detection limit, enabling an enhanced sensitivity of corresponding analytes specific for health and medical conditions under consideration. Research in the development of modified nanobodies as the BRE and microfluidic devices enhances analyte sequestration and provides the tool to amplify the electron transfer rate towards the physical transducer to generate a sensitive, detectable readout. The use of AI has a pivotal role in the development of smart biosensing devices for advanced diagnostics and early detection of the health-to-disease transition [14, 19].

In this EPMA position manuscript, we focus on an emerging aspect in modern healthcare that originates from the technological advancements of our increasingly digitalized world: digital biomarkers. Progressively throughout this report, we will define digital biomarkers, elaborate how they are measured and validated, provide examples on how they transformed multiple indication areas, and explore their utility and potential in the context of predictive, preventive, and personalized medicine (3PM) [20].

## Defining digital biomarkers

**Fig. 1:**
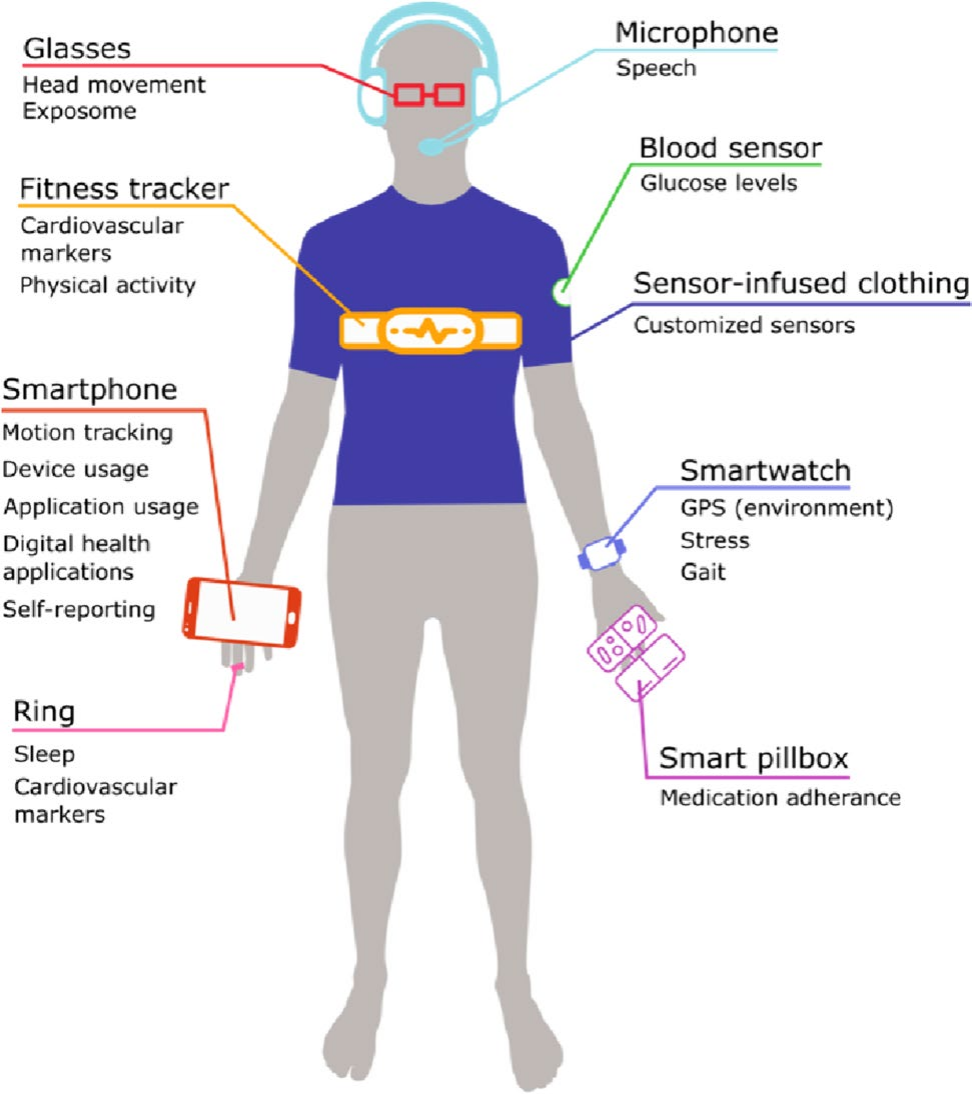
Examples of personalized point-of-care wearable sensors, digital devices, and their applications in healthcare

Digital biomarkers refer to the measurable and quantifiable physiological, behavioral, and environmental parameters collected for an individual through innovative digital health technologies, including wearables, smart devices, and medical sensors. In our modern, digitalized world, human beings constantly interact with digital devices and thereby produce measurable data as schematically presented in Fig. 1. Smartwatches and fitness trackers, for example, measure an individual’s heart rate, physical activity, and resting patterns. Smart devices track how their users interact with specific applications, how long and often they actively use their device throughout the day and are filled with sensors that can recognize subtle movements and record speech. All the measurements mentioned above are well-known modulators of personal health or correlate strongly with it and, thus, it seems obvious that they could support providing optimal healthcare to today’s patients. However, to make them applicable in a healthcare context, they need to be developed from patient-level data and validated to ensure their reliability [21].

Over the recent years, digital health applications have also been developed that specifically aim to monitor an individual’s disease symptoms and progression [22], monitor relevant health indicators in real time and provide notifications if they appear critical, or track dietary habits. Data is provided to these applications through direct transfer from a wearable sensor (e.g., a continuous glucose monitor in the context of diabetes) or can be self-reported by the patient (e.g., tracking mood swings in patients suffering from manic depressions).

## Sleep disorders

Good quality sleep is considered an important aspect of protection against health-to-disease transition (primary care) and against disease progression (secondary care), which makes it instrumental in the context of 3PM. Further, there is evident reciprocity between compromised sleep quality/sleep disorders and a broad spectrum of generally preventable diseases including chronic inflammation; metabolic, immune, and mood disorders; malignancies; cardiovascular disease (CVD); and neurological and neurodegenerative disorders [14, 23–26]. Targeted prevention and individualized monitoring of sleep disorders is crucial to maintain health and life [27–29]. Table 1 highlights the DBs applied and proposed specifically in sleep medicine.

**Table 1:** Examples of DBs used in sleep medicine

Parameters requiring DBM	Description of DB	References
Insomnia	Data from the digital sleep diaries can be applied for the evidence-based cognitive behavioral therapy for insomnia (CBT–I) Following data can be admitted from the digital sleep diaries as digital sleep quality features: total sleep time, sleep latency, and sleep efficacy	[23, 30–33]
Hypersomnia	Data from the digital sleep diaries can be collected using digital wearable devices created to monitor the motor activity of the body (e.g., actigraphy and smart watches) Continuous digital measuring of motor activity for durations of at least 7 days and nights can produce information about the average sleep time and indicate pathological sleep patterns. In addition, digitally validated questionnaires can be used to evaluate daytime fatigue and sleepiness	[34]
Sleep–wake circadian disorders	DBs related to sleep–wake activity are captured through wearable devices such as actigraphy and smart watches. The duration needed for the reliable diagnosis is upwards of 3 weeks. Continuous monitoring of the day-night activity is decisive for detecting sleep–wake irregularities in shift workers and sleep disorders in blind people	[27, 28, 33, 35]
Abnormal light exposure	Light exposure plays a key role in sleep–wake disorders and seasonal depression. Further, light exposure enhances the subjective level of energy. Therefore, several types of wearables and actigraphy devices include light sensors which allow for objective monitoring of the light exposure (dose and day-night rhythms) — information further utilized to improve individual outcomes in the course of depression and sleep disorders	[36]
Obstructive Sleep Apnea (OSA)	The apnea–hypopnea index (AHI), usually measured by polysomnography, is of limited utility but can be enhanced by novel digital devices enabling for multi-parametric measuring of digital oximetry, digital heart rate variability (HRV), digital atrial fibrillation, and digital monitoring of snoring. AI can aid the multi-parametric analysis of validated questionnaires, establishing a comprehensive diagnostic approach and individualize treatment regimes	[37, 38]
HRV	HRV refers to the variation in time intervals between successive heartbeats indicating the level of sympathetic versus parasympathetic activity of the body. HRV is considered an indicator of great clinical utility for monitoring of individualized stress reactions providing insights into sleep arousals and enhanced sympathetic regulations interrupting the sleep architecture which is decisive for a good quality sleep. HRV can be measured using wearable devices with heart rate sensors and via contactless apps	[39]

An increasing number of digital health platforms address the demand on multi-parametric analysis summarized above, and thereby enable professionals to perform digital screenings for sleep disorders, accurate diagnosis, personalized treatments and cost-effective prevention of sleep disorders, and associated pathologies in primary and secondary care.

## Diabetes care

The prominent examples of DBs commonly used in diabetes care and monitoring are presented in Table 2.

**Table 2:** Examples of DBs used in diabetes care

Parameters requiring DBM	Description of DB	References
Blood glucose levels	Continuous glucose monitoring (CGM) systems track glucose levels in real-time using patches (skin-implanted sensors). CGM provides continuous data, allowing individuals and healthcare professionals to monitor glucose levels, identify trends, and make informed decisions about treatment and lifestyle choices. Novel CGM-related metrics, such as time in range, time below range, time above range, glucose variability, glucose management indicator, and average glycemia, have been introduced	[40]
Physical activity	DBs related to physical activity are captured through wearable devices including fitness trackers and smartphones. Parameters monitored include step count, duration of running, distance traveled, active minutes, velocities achieved, and calories burned. Regular physical activity is important for prevention and management of diabetes, and prevention of diabetes complications	[41]
HRV	See above in Table 1	[39]
Sleep patterns	Sleep has been recognized as having a crucial role in diabetes management. Sleep trackers or smart watches monitor sleep patterns, including total sleep time, sleep stages (deep, light, rapid eye movement), and interruptions. By analyzing sleep data, individuals and healthcare providers can identify and moderate factors influencing blood glucose control	[42]
Medication adherence	DBs track medication adherence by monitoring when and if prescribed medications are taken. This is done through smart pill containers, medication reminder apps, insulin pen caps, or electronic health records that log medication administration	[43]
Diet and nutrition	Smartphone apps or wearable devices equipped with sensors can help individuals to monitor their dietary intake. Energy consumption, macronutrient distribution, carbohydrate intake, and meal timing can be tracked. These biomarkers help individuals make informed decisions about food choices and maintain a healthy diet	[44]
Stress levels	Chronic stress can impact blood glucose levels and overall well-being. Wearable devices or smartphone apps can assess stress levels through measures including skin conductance, HRV, and self-reported stress scales. This information can help individuals manage stress and make lifestyle adjustments accordingly	[45]
QoL metrics	DBs capture subjective measures related to QoL, including mood, emotional well-being, and diabetes-related distress. Mobile apps or surveys administered via digital platforms collect this information and provide insights into the psychological impact of diabetes	[46–48]

These DBs enable individuals with diabetes to actively participate in self-management and empower healthcare providers with more comprehensive data for personalized care. Integration of DBs into digital health platforms allows for continuous monitoring, analysis, and early detection of deviations from optimal health. The CGM-related DBs have already become a standard of care in people with both type 1 and type 2 diabetes, resulting in improved outcomes as demonstrated in numerous studies [49–51].

## Cardiovascular diseases

Cardiovascular diseases remain the leading cause of morbidity and mortality worldwide [52]. Traditional approaches to CVD management have primarily relied on subjective patient-reported symptoms and intermittent clinical assessments. Recent advances in digital health technologies have created new possibilities for monitoring and managing CVDs more effectively. Selected DBs related to CVDs are listed in Table 3.

**Table 3:** Examples of DBs used in CVD care

Parameters requiring DBM	Description of DB	References
Electrocardiogram (ECG)	A continuous ECG can be recorded and analyzed in real time to identify numerous anomalies in the cardiac function and rhythm	[53]
HRV	See above in Table 1	[39]
Physical activity and sedentary behavior	Accelerometers and gyroscope sensors integrated into wearable devices track an individual’s physical activity levels and sedentary behavior patterns. Regular exercise has been proven to be beneficial for cardiovascular health, and DBs quantify an individual’s activity levels, allowing for tailored exercise prescriptions and lifestyle interventions	[41]
Blood pressure	Ambulatory blood pressure monitoring through wearable devices provides continuous blood pressure measurements throughout the day. These data help identify individuals with masked hypertension or nocturnal hypertension, aiding in the diagnosis and management of hypertension-related CVDs. Transdermal optical imaging uses advanced machine learning (ML) to predict systolic, diastolic, and pulse pressure from facial blood flow data through contactless apps. It does not require a device and therefore is more convenient and comfortable for users	[54, 55]
Sleep quality assessment	Sleep disturbances, such as sleep apnea and insomnia, are associated with an increased risk of CVDs. Wearable devices equipped with sensors monitor sleep duration, quality, and respiratory patterns, enabling the early identification of sleep-related disorders and timely intervention	[42]

The DBs in CVDs can capture subtle changes in physiological parameters, detect early warning signs, and enable personalized interventions.

## Chronic obstructive pulmonary disease

Chronic obstructive pulmonary disease (COPD) is a progressive respiratory condition characterized by a persistent airflow limitation which leads to significant morbidity and mortality worldwide. The management of COPD traditionally involves subjective symptom reporting and periodic spirometry assessments. DBs offer numerous advantages for COPD management. They enable the detection of minor changes in disease progression, facilitate early intervention, and empower patients to actively participate in their own care. Examples of DBs used in COPD are presented in Table 4.

**Table 4:** Examples of DBs used in COPD care/pulmonary rehabilitation

Parameters and functions requiring DBM	Description of DB	References
Pulmonary function	Spirometry is the gold standard for assessing pulmonary function in COPD patients. However, wearable devices equipped with sensors and algorithms can now capture lung function parameters such as the forced expiratory volume in one second and forced vital capacity outside of the clinical setting. Other wearables detect frequency and strength of breathing using audio of inhalations and expirations together with measurements of chest expansion and contraction. These DBs enable frequent and accurate monitoring of lung function, allowing for timely adjustments in treatment plans	[56, 57]
Physical activity tracking	COPD is often accompanied by reduced physical activity levels which can further exacerbate the condition. Wearable devices with accelerometers can measure parameters such as step count, distance traveled, and energy expenditure. By tracking physical activity patterns, DBs provide insights into an individual’s functional capacity, adherence to exercise programs, and overall disease management	[41]
Oxygen saturation	Oxygen saturation levels are crucial indicators of respiratory function in COPD patients, especially during periods of exacerbation or hypoxemia. Wearable pulse oximeters continuously measure oxygen saturation, alerting individuals and healthcare providers to any fluctuations or abnormalities. This allows for early intervention and the timely adjustment of supplemental oxygen therapy	[58]
Cough	Cough is a prominent symptom in COPD and can be indicative of disease exacerbations or worsening. Smartphone applications and wearable devices equipped with microphones can capture and analyze cough sounds, providing valuable data on cough frequency, intensity, and pattern. This information can aid in assessing disease progression and treatment response	[59]

DBs used in the treatment of COPD have the potential to enhance remote monitoring, reduce hospitalizations, and optimize treatment strategies for COPD patients.

## Cancers

To a large extent, cancer management relies on periodic imaging, laboratory tests, and subjective patient reporting. In contrast, DBs have the potential to revolutionize cancer management across the entire continuum, including real-time monitoring, cost-effective health risk assessment and individualized protection against health-to-disease transition, early detection of disease progression, treatment algorithms tailored to individualized patient profile, response assessment, and improved individual outcomes. Examples of DBs used in cancer management are listed in Table 5.

**Table 5:** Examples of DBs used in cancer management

Parameters and functions requiring DB monitoring	DB description	References
Physical activity and functional status	Wearable devices equipped with accelerometers and other sensors can monitor an individual’s physical activity levels, gait patterns, and functional capacity. Changes in physical activity patterns and functional decline may indicate disease progression, treatment side effects, or general well-being. DBs in this domain offer opportunities for early detection of cancer-related fatigue, functional decline, and personalized rehabilitation programs	[41, 60, 61]
Cancer-related symptoms	Smartphone applications and wearable devices enable individuals to track and report cancer-related symptoms such as pain, nausea, fatigue, and depression. DBs in symptom monitoring provide real-time data on symptom severity, duration, and impact on daily activities. This information facilitates personalized symptom management, early intervention, and improved patient outcomes	[62]
Radiomics and pathomics application	AI algorithms utilize data from digital images from radiology and pathology that are imperceptible to the human eye identifying cancer diagnosis at earlier stages	[63]
Medication adherence	Mobile applications and smart pillboxes can monitor medication adherence, which is crucial for cancer patients undergoing complex treatment regimens. DBs in this area provide insights into patient adherence patterns, missed doses, and potential barriers to medication compliance. This information enables targeted interventions, patient education, and improved treatment outcomes	[64]

## The role of digital biomarkers in predictive medicine

**Fig. 2:**
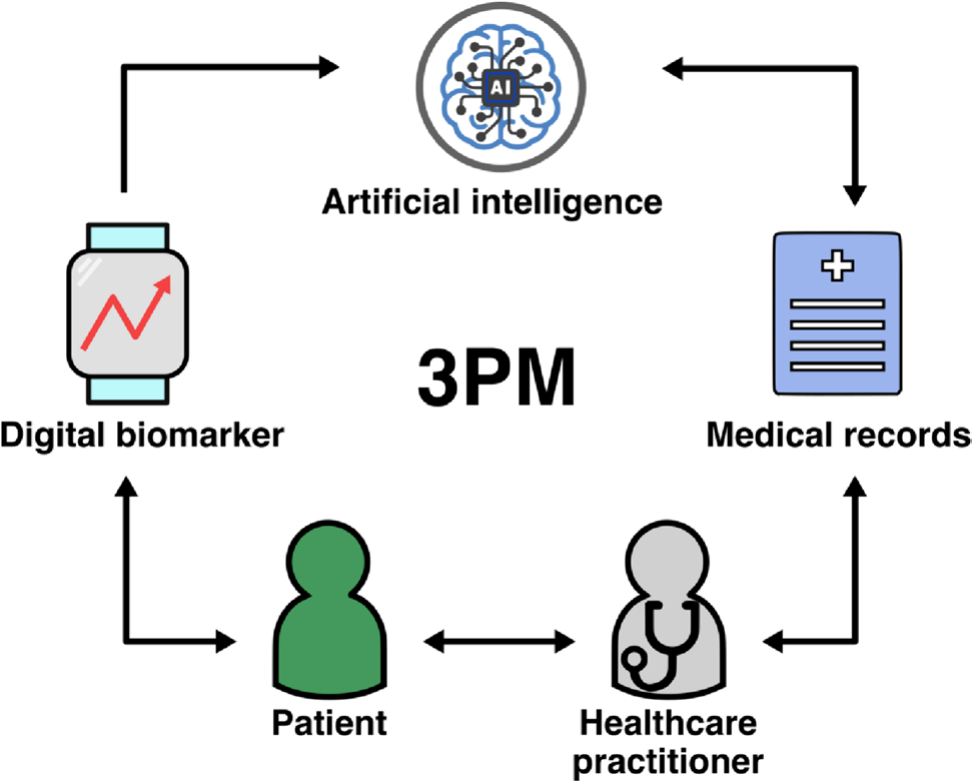
Flow of DB data from wearable to the cloud processed through AI/ML, added to patients’ medical records, and provided to the healthcare practitioner. Previous medical records of the patients can support the AI in interpreting DB patterns. Please note that the patient becomes also aware of DB data increasing its engagement in the management process. Healthcare practitioners are using the DB processed data integrated with other data from the electronic health records (laboratory analyses, imaging, etc.) for adequate management decisions in the frame of 3PM

Digital biomarkers contribute to predictive medicine by providing insights into disease risk, progression, and treatment response. Continuous DBM through wearables, smart devices, or smartphone applications allows patients and healthcare providers to forecast the future progression of NCDs in real time as presented in Fig. 2.

For example, studies have shown CGM to be cost effective, reduce hospitalizations, and improve QoL in patients with diabetes [65, 66], and home-based blood pressure monitoring was found to be more cost-effective compared to clinic-based care [67]. Telehealth could play a role in the care and management of people with COPD, and as part of multi‐component care packages may provide short‐term benefits for QoL and hospital re‐admissions [68]. Remote monitoring systems for COPD are expected to become integrated into the healthcare system, which could reduce costs and improve the quality of care by predicting an individual patient’s response and risk [69].

Considering the strong association between diabetes and CVDs, or diabetes and cancer, integrating several DBs related to different NCDs could enhance their predictive value.

Data-driven approaches from the field of AI and ML can analyze trends and patterns in DBs and predict potential exacerbations, complications, or a patient’s response to specific interventions. Providing healthcare practitioners with this information facilitates and focuses discussions about healthcare evaluation and implementing treatment regimens. The predictions provide decision support to clinicians and allow them to select personalized treatments that optimize therapeutic outcomes while minimizing adverse effects. This also opens the opportunity to take early, proactive measures, such as adjusting medications, recommending lifestyle modifications, or initiating early interventions to prevent adverse health outcomes. Predictions made with AI approaches leveraging DBs can also be utilized to inform screening processes in clinical trials and enrich trial cohorts with specific disease subtypes. This, subsequently, also enables employing novel clinical trial designs, such as umbrella or basket trials [70].

## The role of digital biomarkers in preventive medicine

Prevention plays a crucial role in reducing the burden of NCDs. DBs offer novel opportunities to identify individuals at high risk of developing the condition and implement effective preventive strategies.

Wearable devices, such as smartwatches and fitness trackers, collect real-time data on vital signs, physical activity, sleep patterns, and other physiological parameters. To create an opportunity for early disease prevention, the means for early disease detection or risk prediction must be opened [71]. AI algorithms and ML models are poised to identify patterns and anomalies in an individual’s DB patterns that may signify the early onset of a disease or an increased risk (Fig. 2).

Considering the strong links between individual NCDs (diabetes and CVDs, diabetes and cancer, diabetes and COPD), it is obvious that the same DBs are associated with different NCDs, as demonstrated in the above presented Tables 1, 2, 3, 4, and 5. Hence, the prevention strategies towards individual NCD could benefit other NCDs, as well. Simultaneously, this bears challenges for differential diagnostics based on DBs alone, as subtle differences and interdependencies between them might be crucial to distinguish between related diseases. To this end, advanced AI models leveraging multi-modal datasets could demonstrate potential [72].

By analyzing the DBs used in diabetes care, for example, healthcare practitioners and patients can detect early signs of prediabetes, such as impaired fasting glucose or postprandial hyperglycemia. However, more specific guidelines are needed on the use of CGM metrics in defining people with prediabetes, as a target population for diabetes prevention. DB-related information enables targeted interventions, including lifestyle modifications, personalized nutrition plans, and exercise regimens, to prevent or delay the onset of diabetes. DBs could be of critical importance for the prevention of other NCDs, as well.

## The role of digital biomarkers in personalized medicine

Personalized medicine aims to provide tailored interventions based on an individual’s unique characteristics, including genetics and environmental factors. DBs provide real-time, comprehensive data on an individual’s physiological, behavioral, and environmental parameters, enabling healthcare practitioners to gain a holistic view of a patient’s health and develop precise and personalized care plans (Fig. 2). Adequate management of NCDs is highly individualized, considering the diverse factors influencing the overall health of a patient. An individual with diabetes may benefit from a personalized nutrition plan, exercise regimen, and medication schedule based on its real-time glucose levels, activity patterns, and genetic markers. Likewise, it is important to evaluate the individual genetic markers of a cancer patient to administer the optimal treatment.

DBs and health applications offer additional benefits beyond the individualized planning and administration of treatment regimens by a healthcare practitioner. The patient benefits directly, as devices and apps provide direct, personalized feedback providing an opportunity to enhance patients’ understanding about the consequences of their behaviors, and suggest data-guided behavior change. Furthermore, they can help to monitor and incentivize treatment compliance and provide real-time reminders to patients to take medication. Diabetes patients, for example, can wear measurement patches that continuously monitor their glucose levels and send notifications to a patient’s smartphone should the levels reach critical ranges.

The personalized recommendations can be enabled and enhanced by AI methods. By analyzing the potentially complex patterns emitted through measuring an individual’s DBs, they allow them to make personalized predictions. Consequently, these predictions can be used to offer personalized behavior recommendations, optimize medication regimens, and provide ongoing guidance and support for individuals with NCDs.

## Telemedicine and digital biomarkers

Another area of DB utilization in the light of the 3PM is telemedicine. The potential of telemedicine has been demonstrated during the recent COVID-19 pandemics, and in many areas remote healthcare practices have continued even after the end of the pandemics [73, 74].

In the ever-evolving landscape of healthcare, technology has emerged as a powerful tool to enhance patient care, improve access to services, and optimize resource utilization. Telemedicine, the remote delivery of healthcare services through telecommunications technology, has revolutionized the way patients interact with their healthcare practitioners, offering a convenient and accessible alternative to traditional in-person visits [75]. Coupled with the advent of DBs, telemedicine is poised to transform the future of healthcare delivery. For example, patients with NCDs can be remotely monitored through DBs, allowing their healthcare practitioners to adjust their treatment plan as needed, potentially preventing complications and hospitalizations.

Telemedicine and DBs also play a crucial role in addressing healthcare disparities and improving access to care, particularly in underserved and rural communities. In areas where in-person healthcare practitioners are scarce, telemedicine can bridge the gap, providing patients with essential medical services from the comfort of their homes. The use of telemedicine and remote monitoring also represents an opportunity for the healthcare industry to lower its carbon footprint by reducing carbon dioxide emissions associated with travel to and from clinic-based care.

Moreover, telemedicine and DBs can promote patient engagement and empowerment (Fig. 2). By providing patients with real-time access to their health data, these technologies can foster greater self-awareness and encourage active participation in their healthcare decisions. Patients can track their progress, identify potential concerns, and communicate more effectively with their healthcare practitioners, leading to a more personalized and patient-centered approach to care. As these technologies continue to evolve, their impact on healthcare is bound to grow, paving the way for a more efficient, accessible, and patient-centered healthcare system.

## Challenges and future directions

While the potential of digital biomarkers in the context of 3PM is promising, several challenges need to be addressed. Data privacy and security concerns, regulatory oversight, and standardization of data collection and analysis methods are critical considerations. Additionally, the integration of DBs into routine clinical practice requires healthcare practitioners to be adequately trained in interpreting and utilizing digital health data effectively.

Building predictive AI models that utilize DBs to enable personalized, predictive, and potentially preventive interventions is non-trivial and requires interdisciplinary expertise by leveraging modern computer science concepts and aligning them to medical challenges and regulations. Gaining regulatory approval to apply such models in routine clinical care requires dedicated clinical trials for the technology in question [71, 76]. Looking ahead, advancements in AI and ML algorithms will enable more accurate analysis and interpretation of DB data. Additionally, the integration of multiple data sources, such as genomics, proteomics, and imaging, will further enhance the predictive power of DBs [72].

Despite these challenges, the future of DBs in the management of NCDs appears promising. Ongoing research, collaborations between technology and healthcare sectors, and advancements in AI and ML algorithms are likely to enhance the accuracy, reliability, and clinical utility of DBs.

### Pediatric diseases in focus of future DB-based care

**Fig. 3:**
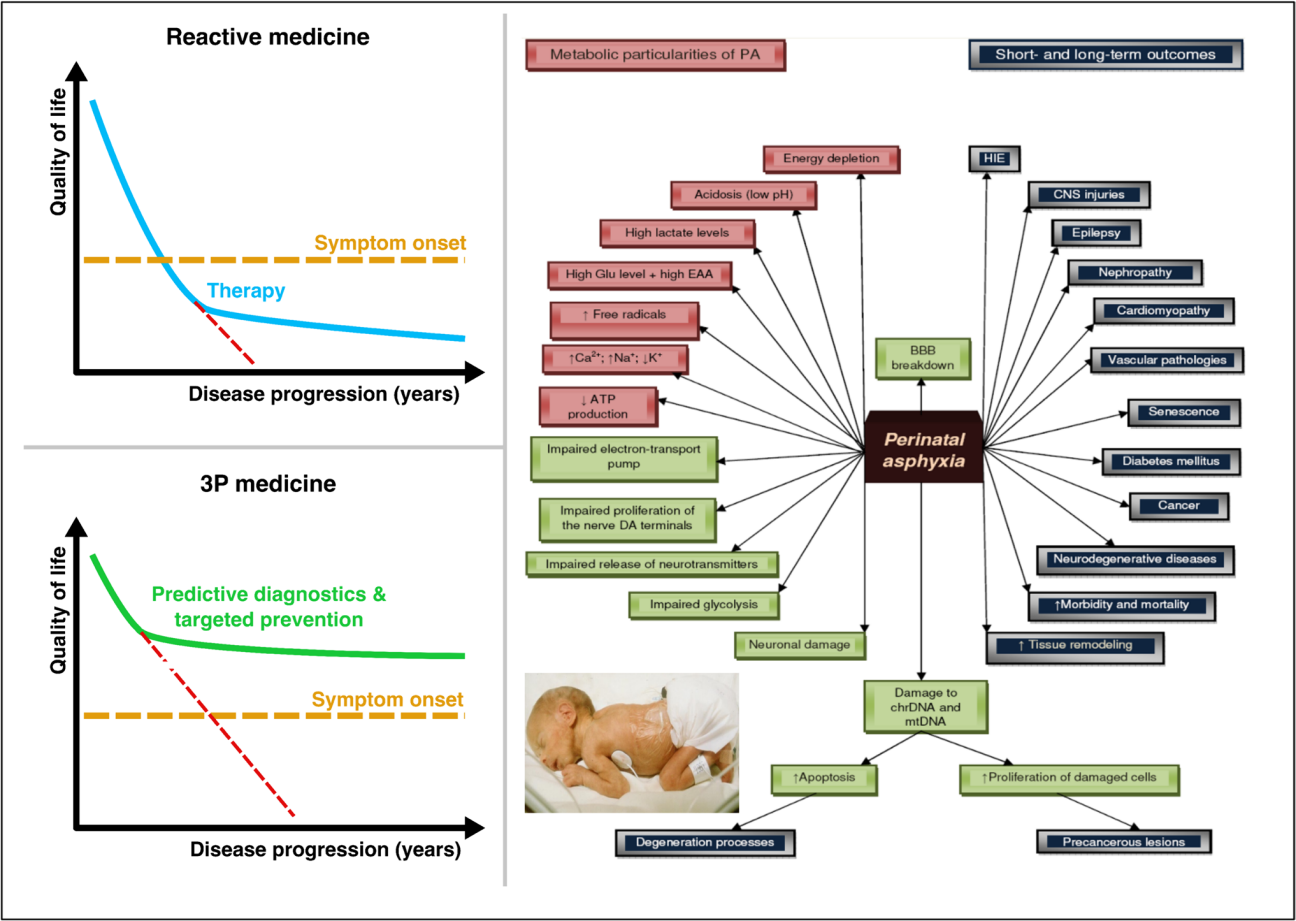
PA as the most frequent medical condition in newborns: concepts of 3PM and point of care for application of DBM. The image is adapted from [20, 77–79]

There are various pediatric diseases in which DBM could be helpful, for example, asthma, which is among the most common chronic diseases of childhood; perinatal asphyxia (PA); lack of oxygen at birth; and a more severe form of PA, hypoxic-ischemia encephalopathy. PA-affected newborns are potentially predisposed to cascading pathologies per evidence including metabolic, neurodegenerative, and malignant diseases [77] as summarized in Fig. 3. Whereas pathologies caused by severe PA are relatively abundantly described, individual health risks linked to mild forms of PA are less well investigated and may act as the trigger for severe pathologies developed later on in life. Corresponding DBM approaches are urgently needed to improve healthcare with significant benefits to the society at large.

Numerous maternal health risk factors are associated with PA, including but not restricted to diabetes, preeclampsia, and uterine (rupture), placental (acute abruption), and cord events (tight nuchal cord). Prepregnancy checkup of maternal vascular status and associated phenotype is crucial for the health of mother and offspring, predictive approach, and targeted prevention [80]. Application of telemedicine is crucial for primary prevention of birth asphyxia, particularly in areas with low density of healthcare units [77]. Application of AI is essential for multi-parametric analysis and effective data interpretation in predicting and preventing PA. The 3PM concepts are summarized in Fig. 3.

## Concluding remarks and outlook in the framework of 3P medicine

In Fig. 1 it is clear that wearables are a key component to DBM. How can we define “wearables”?

“Wearables” are devices, in close contact with the individual, that provide personalized information to guide and optimize prediction, prevention, diagnosis and treatment of conditions affecting the individual’s healthcare and QoL”[81].

Wearables offer two critical advantages for predictive, preventive, and personalized diagnosis and treatment:

1. Wearables provide continuous monitoring, data collection, and treatment. Given that so many human functions and disorders exhibit circadian rhythms and other variations over time, continuous monitoring is essential for accurate personalized diagnosis and treatment.
2. Data from (and interventions by) wearables are transmitted directly to the “cloud.” The advantages of bypassing the need for a patient to visit a lab or clinic or hospital for data collection are several: (a) the logistics or expense for the patient to travel to a healthcare facility can be extremely burdensome if not prohibitive; (b) the time delay if data is collected at the healthcare facility can be fatal (e.g., hypoglycemia or hyperglycemia in diabetes, cardiac arrhythmias); and (c) the limited healthcare personnel in most LMICs (and many HICs as well) make personal visits for data collection impractical if not impossible.

The advantages of wearables plus AI can be linked to predictive, preventive, and personalized medicine [82]:

1. Predictive: gathering continuous data from thousands (or millions) of individuals allows the detection of early signals of future disorders (e.g., prediabetes, early cancer detection).
2. Preventive: once predictive data are available, one can determine the interventions that will be most effective to prevent (or ameliorate) the evolution of early signs into a full-blown disorder — whether activity alterations, dietary changes, and/or medications.
3. Personalized: the individual’s data can be compared with those data from other individuals with similar healthcare backgrounds as well as with “big data” from a population numbering in the thousands if not millions. The most efficacious predictive, preventive, and treatment strategies for that individual can thus be determined.

### Participatory medicine and acceptance in the population

The evolution of digital technology and data science will positively impact healthcare, providing unprecedented opportunities for improving prediction, prevention, and personalization of disease management of individuals affected by NCDs, vulnerable sub-populations, and for society at large. However, citizens’ trust becomes essential for harnessing the full potential of the new digital possibilities and realities in which there would be integration of data of different types (e.g., real-world data and clinical research data) and the use of AI-based medical device software. Participation of citizens is thus the prerequisite for fostering a new collaborative and innovative ecosystem. Currently, there is no full trust on how all the retrieved data are kept, if they are accessed securely, used appropriately and responsibly, without jeopardizing their privacy. Only when this trust is obtained, citizens will be more likely to consent to the sharing and processing their data for secondary and tertiary use in healthcare, research, and innovation.

Developing and implementing transparent and accountable data governance frameworks play a vital role in creating citizens’ trust. To create this trust, it is essential to address any misconceptions, fears, or concerns that the citizens may have about data privacy, security, and the potential misuse of their data. Hence, engagement in open dialogue, providing accessible and transparent information, and promotion of public awareness campaigns are fundamental to contribute to a better understanding of the benefits and risks associated with data sharing and with digital tool development. Furthermore, citizens should be involved in decision-making processes (Participatory medicine). The demonstration of the tangible benefits of the new digital reality, such as improvements in personalized medicine and public health, fosters empowerment, and positively influences the citizen’s perceptions and behavior. By addressing these factors, healthcare practitioners, researchers, and policy makers can create an environment of trust, ultimately leading to increased participation and support for digital health.

## Data Availability

Not applicable.

## Abbreviations

AFP: Alpha-fetoprotein
AHI: Apnea-hypopnea index
AI: Artificial intelligence
BRE: Biorecognition element
CBT-I: Cognitive behavioral therapy for insomnia
CEA: Carcinoembryonic antigen
CGM: Continuous glucose monitoring
COPD: Chronic obstructive pulmonary disease
CVD: Cardiovascular disease
DB: Digital biomarker
DBM: Digital biomarker monitoring
ECG: Electrocardiogram
EEG: Encephalogram
EGFR: Epidermal Growth Factor Receptor
EPMA: European Association for Predictive, Preventive, and Personalized Medicine
HE4: Human epididymis protein 4
HIC: High-income countries
HRV: Heart rate variability
LMIC: Low- and middle-income countries
ML: Machine learning
MUC16: Mucin-16
NCD: Non-communicable chronic disease
OSA: Obstructive sleep apnea
PA: Perinatal asphyxia
PPPM/3PM: Predictive, preventive, and personalized medicine
PSA: Prostate-Specific Antigen
QoL: Quality of Life
TYR: Tyrosinase

## Declarations

### Disclaimer

O. G. is the Editor-in-Chief of the journal, but was not involved in, did not influence over, nor accessed to the details of the peer review process of this work.
